# Innate Immune Surveillance and Recognition of Epigenetic Marks

**DOI:** 10.3390/epigenomes9030033

**Published:** 2025-09-05

**Authors:** Yalong Wang

**Affiliations:** Department of Epigenetics & Molecular Carcinogenesis, The University of Texas MD Anderson Cancer Center, Houston, TX 77030, USA; ywang68@mdanderson.org; Tel.: +1-832-750-7224

**Keywords:** immune recognition, PAMPs, DAMPs, PRRs, epigenetics, viral mimicry, cancer immunotherapy

## Abstract

The innate immune system protects against infection and cellular damage by recognizing conserved pathogen-associated molecular patterns (PAMPs) and damage-associated molecular patterns (DAMPs). Emerging evidence suggests that aberrant epigenetic modifications—such as altered DNA methylation and histone marks—can serve as immunogenic signals that activate pattern recognition receptor (PRR)-mediated immune surveillance. This review explores the concept that epigenetic marks may function as DAMPs or even mimic PAMPs. I highlight how unmethylated CpG motifs, which are typically suppressed using host methylation, are recognized as foreign via Toll-like receptor 9 (TLR9). I also examine how cytosolic DNA sensors, including cGAS, detect mislocalized or hypomethylated self-DNA resulting from genomic instability. In addition, I discuss how extracellular histones and nucleosomes released during cell death or stress can act as DAMPs that engage TLRs and activate inflammasomes. In the context of cancer, I review how epigenetic dysregulation can induce a “viral mimicry” state, where reactivation of endogenous retroelements produces double-stranded RNA sensed by RIG-I and MDA5, triggering type I interferon responses. Finally, I address open questions and future directions, including how immune recognition of epigenetic alterations might be leveraged for cancer immunotherapy or regulated to prevent autoimmunity. By integrating recent findings, this review underscores the emerging concept of the epigenome as a target of innate immune recognition, bridging the fields of immunology, epigenetics, and cancer biology.

## 1. Introduction

The human immune system has evolved intricate mechanisms to distinguish “self” from “non-self” and to detect signs of danger or infection. In innate immunity—the body’s first line of defense—germline-encoded pattern recognition receptors (PRRs) on cells such as dendritic cells, macrophages, and others constantly scan for molecular patterns indicative of invading pathogens or tissue damage. Classical pathogen-associated molecular patterns (PAMPs) are conserved motifs unique to microbes (for example, bacterial lipopolysaccharide or viral double-stranded RNA) that are absent from healthy host tissues. Recognition of PAMPs using PRRs initiates downstream signaling cascades that engage transcription factors such as NF-κB and IRFs, leading to the induction of cytokines and interferons [1,2,3]. In parallel, the immune system also recognizes endogenous alarm signals. Damage-associated molecular patterns (DAMPs, also called alarmins) are host-derived molecules released during cell injury or stress—such as ATP, HMGB1, mitochondrial DNA, or nuclear proteins—which, when exposed extracellularly, signify danger and likewise engage PRRs to provoke inflammation [4,5,6,7]. Together, PAMPs and DAMPs provide critical cues that something is amiss, prompting innate immune cells to respond and, if needed, to activate adaptive immunity.

Epigenetic marks are chemical modifications that regulate gene expression without altering the DNA sequence, primarily through mechanisms such as DNA methylation—typically at cytosine-phosphate-guanine (CpG) dinucleotides in vertebrates—and various post-translational histone modifications, including methylation and acetylation of histone tails. These marks function as intracellular regulatory signals that influence chromatin structure and gene transcription [8,9], and are normally sequestered within the nucleus, hidden from immune detection. However, an emerging question is whether certain epigenetic alterations might be recognized by the immune system as indicators of cellular stress or “altered-self” [10,11,12]. In other words, can epigenetic marks serve as DAMPs or PAMP-like signals that trigger innate immune recognition? This concept challenges the classical view of immunosurveillance and suggests that the immune system may also monitor epigenomic changes as part of its broader defense strategy.

Several lines of evidence hint at a connection between epigenetic anomalies and immune pattern recognition. For instance, vertebrate DNA is typically methylated at CpG motifs, whereas bacterial and viral DNA often is not; the endosomal receptor TLR9 exploits this difference by selectively recognizing unmethylated CpG DNA and initiating MyD88-dependent signaling that activates NF-κB and IRF7, culminating in the expression of type I interferons and pro-inflammatory cytokines [13,14]. However, aberrant hypomethylation of host DNA (such as in cancer cells or mitochondrial DNA) can similarly lead to TLR9 activation, blurring the line between PAMP and DAMP [15]. Likewise, chromatin or nucleosomes released from dying cells—which carry histone proteins with various modifications—have been shown to activate Toll-like receptors (e.g., TLR2, TLR4, and TLR9) and the NLRP3 inflammasome, functioning as potent DAMPs in sterile inflammation [16,17,18]. Furthermore, emerging research in oncology indicates that disrupting tumor epigenetic regulators can provoke an antiviral-like immune response: demethylation of tumor DNA may awaken silent endogenous retroviruses, leading to double-stranded RNA (dsRNA) accumulation and interferon release—a phenomenon termed “viral mimicry” [19,20]. These examples suggest that, under certain conditions, the immune system can sense epigenetic alterations as signals of infection or danger.

In this review, I focus on human systems to explore how innate immune cells might detect epigenetic alterations and the implications of such recognition for disease, particularly cancer. I begin by summarizing the molecular mechanisms through which PRRs detect epigenetic marks or their downstream consequences, emphasizing key receptors such as Toll-like receptors (TLRs), NOD-like receptors (NLRs), AIM2-like receptors (ALRs), RIG-I-like receptors (RLRs), and the cGAS-STING pathway. I then examine evidence from cancer models demonstrating how epigenetic dysregulation can either activate immune responses or facilitate immune evasion. To contextualize these findings, I analyze the parallels and distinctions between classical PAMPs/DAMPs and epigenetic signals, highlighting how “altered-self” cues may mimic or diverge from traditional non-self or damage signals in immune activation. Finally, I address key unanswered questions and future research directions, including the therapeutic potential of targeting these pathways and the risk of autoimmune responses if the immune system misinterprets epigenetic changes as pathogenic. Through this lens, I aim to provide a comprehensive overview of immune surveillance of the epigenome and its emerging significance in health and disease.

## 2. Epigenetic Alterations as Danger Signals: Conceptual Framework

Epigenetic modifications refer to a diverse range of chemical marks on DNA, RNA, and chromatin that regulate gene expression without altering the underlying genetic sequence. In healthy cells, these patterns are tightly controlled to maintain genomic stability and cellular identity. However, in pathological contexts such as cancer or viral infection, epigenetic regulation can become profoundly disrupted. For instance, cancer is frequently associated with global DNA hypomethylation, promoter-specific hypermethylation, and aberrant histone modifications [21,22], while viruses may hijack host epigenetic machinery to enhance replication or evade immune detection [23].

Traditionally, “danger signals” refer to molecules such as ATP or HMGB1 that are released from damaged or dying cells to alert the immune system [5,7,24]. Here, I propose that epigenetic alterations may constitute a novel class of immunological cues. Through analogy to PAMPs and DAMPs, I conceptualize epigenetic alteration–associated molecular patterns (EAMPs) as a third category of immunostimulatory signals. While PAMPs arise from non-self microbial components and DAMPs from mislocalized or excessively abundant host molecules, EAMPs arise from dysregulated epigenetic states—typically occurring under conditions of stress or pathology—that give rise to aberrant molecular patterns recognized using PRRs as indicators of “altered-self” or cellular dysfunction.

To illustrate this conceptual framework, Table 1 compares traditional PAMPs, DAMPs, and EAMPs in terms of their sources and PRR recognition. In the following sections, I examine the molecular pathways and PRRs involved in sensing epigenetic alterations and their implications in health and disease.

## 3. Molecular Mechanisms of Epigenetic Alteration Recognition by PRRs

Innate immune cells utilize a repertoire of PRRs to detect molecular patterns. Here, I detail how specific PRRs can sense epigenetic modifications or their downstream effects, converting an epigenetic aberration into an immune-activating signal.

### 3.1. Sensing Unmethylated DNA Patterns

A well-established example of epigenetic regulation influencing immune recognition is CpG DNA methylation [9]. In vertebrates, CpG methylation is a key epigenetic modification involved in gene regulation, development, and genomic stability. Vertebrate genomes exhibit widespread CpG methylation, with exceptions like CpG islands—often near gene promoters—that remain unmethylated to permit transcription factor binding and gene expression. In contrast, microbial genomes, including those of bacteria and DNA viruses, are typically enriched in unmethylated CpG-rich motifs [9,33]. TLR9, located in the endosomes of innate immune cells, has evolved to detect these microbial signatures. Upon recognizing unmethylated CpG DNA, TLR9 undergoes proteolytic activation and signals through the MyD88 pathway, leading to activation of IRF7 and NF-kB and the subsequent induction of type I interferons and pro-inflammatory cytokines [13,14].

While TLR9 primarily senses microbial DNA, it can also respond to hypomethylated self-DNA under pathological conditions. During necrosis, stress, or defective clearance, endogenous DNA may access endosomes—particularly via phagocytosis—and, if hypomethylated, it can activate TLR9 similarly to pathogen-derived DNA. Moreover, mitochondrial DNA, which is inherently CpG-rich and hypomethylated, is a known DAMP that activates TLR9 and contributes to systemic inflammation. Likewise, hypomethylation associated with cancer and aging may produce immunogenic DNA fragments—such as microsatellite or retroelement DNA—that stimulate immune responses via TLR9 [34,35].

In parallel, the cytosolic DNA sensor cGAS detects double-stranded DNA independent of sequence and catalyzes cGAMP synthesis to activate STING, triggering the production of type I interferons and pro-inflammatory cytokines [36,37]. Evidence suggests that unmethylated or hypomethylated DNA may more potently activate cGAS [38]. Interestingly, recent studies have shown that endogenous cGAS is predominantly localized in the nucleus, where it is tightly tethered to chromatin [39,40]. Despite its proximity to abundant nuclear DNA, cGAS remains inactive in the nucleus due to inhibitory interactions with nucleosomes, particularly histones H2A–H2B, which block its DNA-binding interface and prevent activation. This sequestration mechanism is essential to prevent inappropriate activation of cGAS by self-DNA and maintain immune tolerance. In autoimmune diseases like lupus, persistent self-DNA—whether in endosomes or cytosol—can simultaneously engage TLR9 and cGAS, promoting chronic inflammation [32].

Thus, the epigenetic status of DNA—specifically CpG methylation—plays a critical role in determining whether DNA is perceived as self or non-self. Loss of nuclear integrity or methylation can convert self-DNA into a perceived danger signal for both endosomal and cytosolic DNA-sensing PRRs.

### 3.2. Extracellular Chromatin and Histones as DAMPs

Under physiological conditions, nuclear chromatin components are compartmentalized and shielded from immune surveillance. However, during necrosis, apoptosis, or neutrophil extracellular trap (NET) formation, DNA and histones can be released into the extracellular environment [41,42]. Histones—highly cationic proteins subject to diverse epigenetic modifications—are inherently cytotoxic and immunostimulatory, capable of disrupting cellular membranes and engaging PRRs.

Once extracellular, histones and nucleosomal DNA activate multiple innate immune pathways. Histones H3 and H4, enriched in positively charged residues, bind to TLR2 and TLR4, initiating MyD88-dependent NF-κB activation and the subsequent release of pro-inflammatory cytokines such as TNF-α and IL-6 [18]. In vivo studies demonstrate that histone administration elicits TLR2/4-mediated inflammation and organ injury, implicating these receptors in sepsis and acute tissue damage. In parallel, TLR9—typically recognizing unmethylated CpG DNA—is also activated by histone–DNA complexes within NETs, driving type I interferon production in plasmacytoid dendritic cells [31]. Chromatin fragments may facilitate TLR4 internalization and subsequent TLR9 engagement, while histone-bound DNA can also upregulate adhesion molecules (e.g., E-selectin, ICAM-1, and VCAM-1) in endothelial cells via TLR9 signaling [10]. In the cytosol, internalized histones trigger activation of the NLRP3 inflammasome in macrophages and dendritic cells, leading to caspase-1–dependent maturation of IL-1β and IL-18 [43]. This inflammasome activation typically requires TLR-mediated priming and is further enhanced by histone-induced reactive oxygen species (ROS). In murine models, NLRP3 deficiency mitigates histone-induced inflammation, and in liver injury, histones activate Kupffer cell inflammasomes via a TLR9-ROS-dependent pathway [44].

Emerging evidence suggests that specific histone modifications can modulate their immunogenic potential or clearance [45,46]. For example, citrullination of histone H3 by peptidylarginine deiminase 4 (PAD4) during NETosis promotes chromatin decondensation and extracellular release [47,48]. Citrullinated H3 is also a well-established autoantigen in autoimmune diseases [49]. While it remains unclear whether PRRs can directly distinguish between specific histone modifications, such alterations may enhance chromatin exposure and facilitate immune activation.

**Figure 1:**
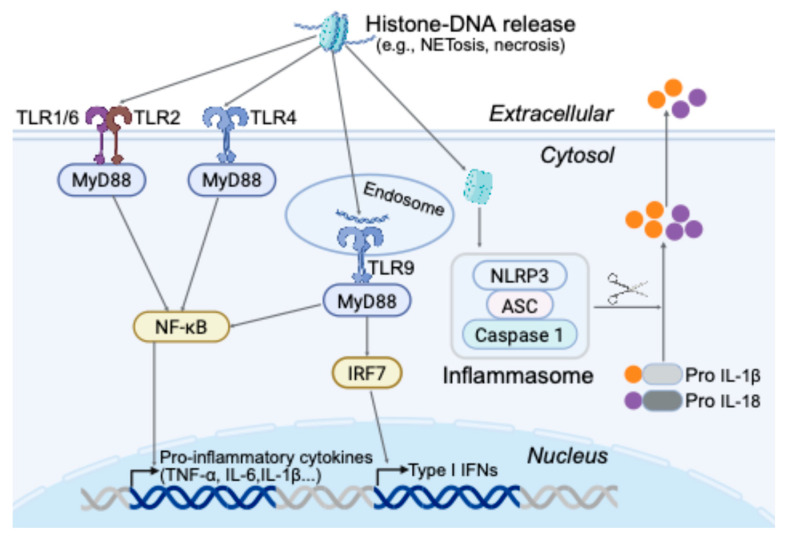
Pathways through which extracellular histones and DNA act as DAMPs to stimulate innate immunity. Histone–DNA complexes released during cellular stress, trauma, or cell death act as potent danger-associated molecular patterns (DAMPs) that activate innate immune responses. Histones bind to Toll-like receptors TLR4 or TLR2 on immune cells, initiating MyD88-dependent signaling and NF-κB activation, which promotes the expression of proinflammatory cytokines such as TNF-α, IL-6, IL-8, and IL-1β. Histone–DNA fragments internalized into endosomes can activate TLR9, especially when containing hypomethylated CpG motifs, resulting in the production of additional proinflammatory cytokines and type I interferons. In the cytosol, internalized histones can also activate the NLRP3 inflammasome in macrophages and dendritic cells, leading to caspase-1–dependent maturation and secretion of IL-1β and IL-18.

In summary, extracellular chromatin serves as a potent trigger of innate immunity. Histone–DNA complexes released during cellular stress or death engage multiple PRR pathways—including TLR2/4 (membrane), TLR9 (endosomal), and NLRP3 (cytosolic)—ensuring that nuclear content release is recognized as a key danger signal (Figure 1). Future studies investigating histones bearing defined epigenetic modifications under pathological conditions may reveal novel DAMP-like functions, further expanding our understanding of epigenetic contributions to innate immune recognition.

### 3.3. Epitranscriptomic Modifications and RNA Sensing

**Figure 2:**
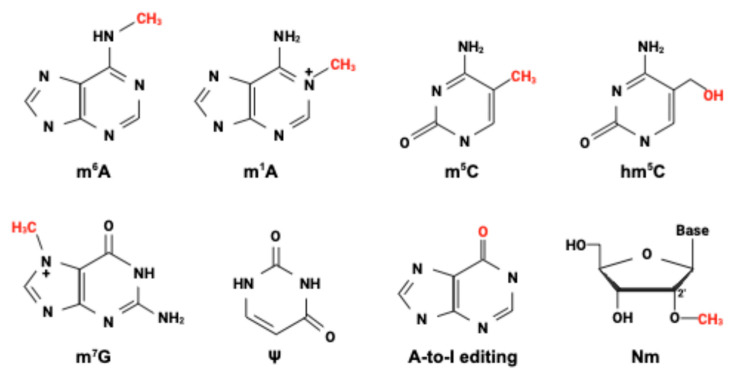
Chemical structures of eight representative RNA modifications. Shown are m^6^A (N^6^-methyladenosine), m^1^A (N^1^-methyladenosine), m^5^C (5-methylcytosine), hm^5^C (5-Hydroxymethylcytosine), m^7^G (7-methylguanosine), ψ (pseudouridine), A-to-I editing (adenosine-to-inosine RNA editing), and Nm (2′-O-methylation, where N denotes any nucleotide).

Just as DNA modifications influence immunorecognition, RNA modifications—collectively referred to as “epitranscriptomic” marks [50,51]—also shape how RNA is perceived using PRRs. More than 170 distinct types of RNA modifications have been found to date, and more will likely be identified. A representative subset of eight RNA modifications is shown in Figure 2. In a seminal paper, Karikó et al. analyzed the activation of TLR3, 7, and 8 through different modified RNAs [52]. Specifically, m^6^A, m^5^C, and ψ modifications were shown to impede stimulation of TLR7 and TLR8. Another well-characterized example involves the RIG-I-like receptors: RIG-I, which detects 5′- triphosphate RNA, and MDA5, which senses long double-stranded RNA in the cytosol [27]. Host RNAs are canonically capped with a m^7^G at the 5′ end and often carry abundant internal modifications such as m^6^A or ψ, which together reduce aberrant recognition by PRRs. In contrast, many viral RNAs lack these protective features, rendering them more susceptible to innate immune sensing.

Of the hundreds of different types of RNA modifications, m^6^A is the most abundant on mRNA and has been a major focus in recent studies. Globally, m^6^A accounts for ~0.1–0.6% of all adenosines and occurs at an average of 3–5 sites per transcript, decorating roughly 25–30% of cellular mRNAs [53]. These modifications play central roles in RNA splicing, translation, and stability, and critically help distinguish self from non-self. Recent studies demonstrate that the quantitative presence or absence of m^6^A strongly influences the viral RNA immunogenicity [54,55,56]. Interestingly, SARS-CoV-2 hijacks the host m^6^A methyltransferase METTL3 to methylate its RNA, thereby suppressing RIG-I activation and delaying interferon responses [57]. Conversely, depletion of m^6^A marks enhances RIG-I recognition and amplifies IFN production. Thus, m^6^A functions as a quantitative “self” signature: abundant in host RNA, scarce or absent in viral RNA. This foundational insight enabled the design of current mRNA vaccines, which incorporate pseudouridine (and occasionally m^6^A enrichment) to increase transcript stability while minimizing innate immune activation.

In summary, epitranscriptomic modifications provide a quantitative and contextual baseline for immune self vs. non-self discrimination. While PRRs do not appear to directly recognize specific modifications, the absence of expected marks renders RNA immunostimulatory. This is analogous to CpG methylation and TLR9: the receptor does not detect the methyl group itself, but instead responds to unmethylated motifs that signal foreign origin. Similarly, RIG-I does not bind m^6^A directly, but unmodified RNA often retains triphosphate ends or forms duplex structures—features absent in properly processed host RNA—triggering immune activation.

An intriguing interface between epigenetics and RNA sensing involves the reactivation of endogenous retroviruses (ERVs) [19,58,59]. Normally silenced via DNA methylation and repressive histone marks, ERVs can become transcriptionally active upon epigenetic de-repression. The resulting double-stranded RNA transcripts are sensed as non-self, primarily by MDA5, triggering type I interferon responses [60]. This mechanism is beneficial for anti-tumor immunity via viral mimicry (as discussed in Section 4) but can also provoke unwanted inflammation. Similarly, loss of RNA-editing enzymes like ADAR1, which deaminates adenosines in dsRNA, leads to accumulation of unedited, immunogenic dsRNA derived from repetitive elements [61]. This activates MDA5 and PKR, mimicking a viral infection and highlighting how loss of post-transcriptional regulation can initiate innate immune responses against self.

In conclusion, RNA modifications—like their DNA counterparts—serve as critical molecular cues for innate immune discrimination. By embedding abundant marks such as m^6^A, host cells define their transcripts as self, while unmodified or aberrantly edited RNAs serve as potent danger signals for PRRs, including RIG-I, MDA5, TLR7, and TLR8. The innate immune system thus integrates not only sequence and structure but also the quantitative modification landscape of RNA to distinguish friend from foe.

## 4. Evidence from Cancer Models and Disease Contexts

The relationship between epigenetic alterations and immune recognition is especially pertinent in cancer. Cancer cells frequently harbor aberrant epigenetic landscapes—global DNA hypomethylation, locus-specific hypermethylation, and dysregulated histone modifications—which can both promote tumor growth and render the tumor immunologically distinct to the immune system. The concept of cancer immunosurveillance traditionally revolves around T cells recognizing mutated antigens; however, innate immune detection of altered molecular patterns in tumors is an emerging paradigm. Here, I highlight evidence and examples from cancer biology where the immune system detects epigenetic dysregulation as a cue, with a focus on human-relevant findings.

### 4.1. Viral Mimicry in Tumors: Endogenous Retroviruses and Interferon Activation

One of the most compelling demonstrations that epigenetic alterations can trigger innate immune responses in cancer is the phenomenon of “viral mimicry” [19,62]. This occurs when the epigenetic silencing of repetitive genomic elements—particularly endogenous retroviruses (ERVs) and other transposable elements—is disrupted, leading to aberrant expression of double-stranded RNAs (dsRNAs) that mimic viral infection [19]. Tumors, especially at advanced stages or after treatment with DNA-demethylating agents, often exhibit global DNA hypomethylation. This loss of repression reactivates normally silenced ERVs, resulting in the production of dsRNAs (e.g., from complementary sense and antisense ERV transcripts), which are recognized using cytosolic RNA sensors RIG-I and MDA5 [27]. These sensors activate the MAVS adaptor protein and downstream IRF3/7 and NF-κB pathways, culminating in the production of type I and III interferons. The resulting interferon response induces an anti-tumor immune state—upregulating MHC class I expression, recruiting dendritic cells, and enhancing T cell cross-priming against tumor antigens.

This concept has been translated into therapeutic strategies. Epigenetic agents such as DNA methyltransferase inhibitors (DNMTis; e.g., 5-azacytidine and decitabine) and histone deacetylase inhibitors (HDACis) can deliberately induce viral mimicry in cancer cells [63,64]. In ovarian cancer and melanoma models, DNMT inhibition has been shown to upregulate ERV and retrotransposon transcription, resulting in intracellular dsRNA accumulation and activation of interferon-β and interferon-stimulated genes (ISGs) via the MDA5-MAVS axis. A landmark study by Chiappinelli et al. [65] first demonstrated this in ovarian cancer, showing that 5-azacytidine treatment induced a “viral defense” gene signature through re-expression of ERVs. Subsequent studies confirmed that combining epigenetic therapy with immune checkpoint blockade yields synergistic effects: epigenetic therapy can convert immunologically “cold” tumors into “hot” ones by driving interferon responses and T cell infiltration [66,67].

Viral mimicry exemplifies how the immune system can be activated not using exogenous pathogens but via altered self, through the dysregulation of endogenous genomic elements. It highlights how a canonical PAMP—dsRNA—can originate from within the host genome under epigenetic stress. Clinically, this paradigm is being explored through trials combining low-dose DNMTis with checkpoint inhibitors to enhance anti-tumor immunity by promoting interferon signaling and T cell recruitment.

Notably, there are regulatory feedback mechanisms that modulate this response. One such mechanism involves the RNA-editing enzyme ADAR1, which deaminates adenosines in dsRNA to prevent immune activation [61]. Loss of ADAR1 leads to the accumulation of unedited, immunostimulatory dsRNA that potently activates MDA5. While this can result in inflammatory toxicity in normal tissues, in cancer cells, ADAR1 deficiency may enhance immune recognition and therapeutic response by increasing their visibility to innate sensors.

### 4.2. DNA Damage, Micronuclei, and cGAS-STING in Cancer

Beyond RNA, DNA-sensing pathways also play a critical role in immune recognition of cancer. Many tumors exhibit genomic instability—accumulating DNA damage, double-strand breaks, mis-segregated chromosomes, and micronuclei [68,69]. These micronuclei, small extranuclear bodies containing chromosomal fragments, are prone to rupture, releasing DNA into the cytosol. Cytosolic DNA activates the cGAS-STING pathway either within tumor cells or in phagocytic host cells that engulf tumor debris [70]. This activation induces type I interferon production and chemokines such as CXCL10, which recruit immune cells to the tumor microenvironment. In murine models, tumors with high chromosomal instability generate stronger innate immune responses through this mechanism, slowing tumor progression when immune effector cells are sufficiently engaged [71].

Therapeutic interventions that increase tumor DNA damage can potentiate cGAS-STING–mediated immunity. Radiation and DNA-damaging chemotherapies—including DNA demethylating agents and topoisomerase inhibitors—can induce micronuclei formation and cytosolic DNA accumulation [72]. For example, inhibition of TTK kinase, a key spindle checkpoint regulator, leads to cytosolic DNA accumulation in hepatocellular carcinoma cells and activates STING, resulting in a senescence-associated secretory phenotype (SASP) that includes chemokines recruiting NK and T cells [73,74]. In breast cancer, paclitaxel-induced micronuclei were shown to activate cGAS [75]; however, chronic STING signaling paradoxically led to upregulation of PD-L1 and pro-tumor cytokines like IL-6—suggesting that while acute cGAS-STING activation may be immunostimulatory, chronic activation may drive immune evasion or tumor-promoting inflammation [76].

Human tumors display heterogeneous STING pathway activity [70]. Tumors with intact cGAS-STING signaling often exhibit spontaneous T cell infiltration and inflammatory gene signatures, correlating with “hot” tumor phenotypes that respond favorably to immunotherapy. In contrast, some tumors evade immune surveillance by silencing this pathway through mutations or transcriptional repression of cGAS or STING. Loss-of-function mutations or epigenetic silencing of STING pathway components have been reported in various cancers, suggesting that immune pressure may select for tumor cells that escape cytosolic DNA detection. Conversely, tumors with high microsatellite instability tend to retain STING activity and are often more responsive to checkpoint inhibitors due to innate immune sensing of DNA damage.

Importantly, tumor DNA can also be sensed using host immune cells. Dying tumor cells—whether through therapy-induced apoptosis or necrosis—release DNA and nucleosomes that are taken up by dendritic cells and macrophages. Within these phagocytes, DNA in phagosomes may activate TLR9, while DNA that escapes into the cytosol can trigger cGAS or the AIM2 inflammasome. Plasmacytoid dendritic cells in the tumor microenvironment have been shown to produce IFN-α upon encountering tumor-derived DNA, especially when complexed with protein. This local interferon production supports recruitment and priming of conventional dendritic cells and T cells against tumor antigens.

An additional example of epigenetic dysregulation promoting immune recognition is the aberrant expression of cancer-testis antigens (CTAs) [77]. Normally restricted to immune-privileged sites such as the testis, CTA genes become demethylated and transcriptionally active in many cancers. Although recognition of CTAs is mediated by the adaptive immune system, it illustrates how epigenetic dysregulation can expose otherwise hidden antigens to T cell surveillance. Innate immunity may also contribute to tumor cells expressing CTAs that can release nucleic acids upon death, activating PRRs such as TLRs and cytosolic sensors, which in turn enhance cross-presentation of CTAs by dendritic cells.

### 4.3. Immunogenic Cell Death and Epigenetic Modulation

When cancer treatments eliminate tumor cells, the mode of cell death significantly influences immune activation. Immunogenic cell death (ICD) is defined by the release of DAMPs such as ATP, HMGB1, and nucleic acids, which engage PRRs on dendritic cells, effectively converting cell death into a vaccine-like event [78]. Epigenetic therapies can promote ICD under certain conditions. For instance, histone deacetylase (HDAC) inhibitors can induce cellular stress that results in surface exposure of calreticulin and release of HMGB1 and ATP—ligands for dendritic cell receptors such as P2 × 7 (for ATP) and TLR4 (for HMGB1) [79,80]. Concurrently, these agents may demethylate tumor DNA, enhancing its visibility to innate immune sensors. Thus, epigenetic therapy exerts a dual effect: generating intracellular viral mimicry and promoting extracellular immunogenic cell death.

Conversely, tumors frequently exploit epigenetic mechanisms to evade immune recognition. Many cancers hypermethylate promoter regions of genes essential for antigen presentation, including components of the MHC class I pathway, or of cytokines required for immune cell recruitment [81]. In addition, key PRR signaling genes can be epigenetically silenced [67]. For example, the chemokine CXCL9, which plays a critical role in T cell recruitment, is often repressed by DNA methylation in immunologically “cold” tumors—an effect that can be reversed by DNA methyltransferase inhibitors, enhancing immune infiltration. Similarly, genes involved in the TLR3 and STING pathways have been shown to be downregulated by DNA methylation in certain cancers, impairing the tumor’s capacity to detect intracellular dsRNA or DNA and initiate an interferon response. This represents a potential immune evasion strategy designed to avoid triggering innate immune activation.

Taken together, cancer offers a unique “natural experiment” for studying the immune system’s response to epigenetic dysregulation. Tumor cells undergo extensive epigenetic reprogramming, generating both pro-immunogenic cues—such as viral mimicry and DAMP release—and immune-suppressive changes, including silencing of key immune-related genes. Therapeutically, tipping this balance in favor of immune activation using epigenetic modulators is a promising approach. While clinical validation of this strategy is ongoing, preclinical models consistently demonstrate that combining epigenetic reprogramming with immunotherapy can render previously evasive tumors susceptible to immune attack. This paradigm highlights how targeted alteration of the tumor epigenome can generate “danger signals” that mobilize innate immune responses against cancer.

## 5. Can Epigenetic Readers Function as Pattern Recognition Receptors?

Epigenetic readers are broadly defined as proteins that recognize covalent modifications on DNA, RNA, or proteins to regulate gene expression [81]. With the growing catalog of epigenetic marks and their corresponding reader proteins, an intriguing question emerges: could certain epigenetic readers also function as non-canonical pattern recognition receptors (PRRs)?

Canonical PRRs detect PAMPs/DAMPs to initiate innate immune responses. In contrast, epigenetic readers typically bind self-derived modifications within chromatin. However, under conditions such as cellular stress, infection, or malignant transformation, epigenetic landscapes can change, and modified nucleic acids or proteins may become mislocalized to the cytosol or extracellular space, where they acquire immunogenic potential. In such contexts, certain reader proteins may contribute to immune surveillance. For example, Tudor domain–containing proteins that bind methylated arginines have been implicated in cytoplasmic signaling [82], while YTH domain proteins that recognize m^6^A on RNA regulate the stability of antiviral transcripts [83]. Though not classical PRRs, their involvement in immune modulation suggests functional convergence. Moreover, innate sensors like AIM2 contain nucleic acid–binding domains structurally similar to those of chromatin readers, pointing to potential evolutionary parallels [84].

From another perspective, certain DNA modifications—such as 6-methyladenine (6mA) [85] and hypermodified 5-hydroxymethylcytosine (5hmC) [86]—are largely absent from mammalian genomes but prevalent in bacterial and viral DNA. These evolutionarily conserved, pathogen-specific marks may act as “non-self” signatures. It is plausible that host cells express reader proteins capable of recognizing such modifications and initiating immune responses. Identifying such proteins would strongly support the concept that epigenetic marks themselves can serve as PAMP-like or DAMP-like signals, expanding the scope of innate immune recognition beyond traditional paradigms.

## 6. Conclusions and Perspectives

Recent discoveries have expanded the scope of immunosurveillance to include monitoring of the epigenome. Epigenetic dysregulation—such as the exposure of unmethylated CpG DNA, mislocalized nucleosomes, or aberrant double-stranded RNAs—can act as immunological alarm signals. These patterns, often resulting from infection, stress, or transformation, mimic classical PAMPs and DAMPs by appearing in abnormal cellular contexts and activating innate immune pathways.

What distinguishes epigenetic signals is their conditional nature. Unlike bacterial lipopolysaccharide or extracellular ATP, epigenetic marks are typically intracellular, reversible, and not inherently immunogenic. Their immune activation potential often depends on mislocalization or functional consequences, such as the generation of immunostimulatory nucleic acids. This context-dependence underscores the immune system’s ability to interpret not just molecular structure, but also spatial and temporal cues.

Clinically, epigenetic-immune crosstalk offers both therapeutic promise and challenges. In cancer, exploiting viral mimicry or chromatin release through epigenetic drugs can boost immunogenicity and synergize with immunotherapies. Conversely, in autoimmunity, aberrant epigenetic states may lower the threshold for inappropriate PRR activation. Understanding how to modulate this balance could guide new interventions.

Key questions remain. What prevents immune activation in the face of minor epigenetic changes during aging? Can innate sensors directly recognize specific modifications like 5hmC or acetylated histones? How do tolerance mechanisms distinguish harmless from dangerous epigenetic patterns?

In summary, epigenetic changes can act as danger signals when misregulated or misplaced, expanding the traditional definition of immune surveillance. By elucidating how innate immunity perceives these alterations, we can better understand and manipulate immune responses in cancer, infection, and autoimmune disease.

## Figures and Tables

**Table 1:** Parallels and distinctions between PAMPs, DAMPs, and EAMPs.

Category of Signal	Source	Recognized by PRRs (Examples)
Pathogen-Associated Molecular Patterns (PAMPs)	Microbial molecules	TLR4 recognizes LPS on bacteria [25]. TLR5 recognizes flagellin [26]. RIG-I/MDA5 sense viral RNA [27]. TLR9 recognizes microbial DNA [14].
Damage/Danger- Associated Molecular Patterns (DAMPs)	Host molecules released due to damage/danger	TLR4 and RAGE bind HMGB1 [28]. P2 × 7 receptor senses ATP [29]. NLRP3 inflammasome is activated by diverse DAMP-induced stress [30].
Epigenetic alteration– Associated Molecular Patterns (EAMPs)	Epigenetic modifications that produce abnormal or pathogen-mimicking patterns without altering the DNA sequence.	TLR9 can respond to host DNA containing unmethylated CpG motifs (normally suppressed by methylation) [31]. extracellular DNA–Histone complexes engage TLR2/4 [18]. cGAS detects any cytosolic DNA (self or viral) leading to STING activation [32].

## Data Availability

Not applicable.
